# Risk assessment of temperature and air pollutants on hospitalizations for mental and behavioral disorders in Curitiba, Brazil

**DOI:** 10.1186/s12940-020-00606-w

**Published:** 2020-07-06

**Authors:** Iara da Silva, Daniela Sanches de Almeida, Elizabeth Mie Hashimoto, Leila Droprinchinski Martins

**Affiliations:** 1 Federal University of Technology – Paraná, Av. dos Pioneiros, 3131, Londrina, PR 86036-370 Brazil; 2 State University of Maringa, Av. Colombo, 5790 - Vila Esperança, Maringá, PR 87020-900 Brazil

**Keywords:** Air quality, Mental diseases, Distributed lag non-linear model, Relative risk

## Abstract

**Background:**

Extreme ambient temperatures and air quality have been directly associated with various human diseases from several studies around the world. However, few analyses involving the association of these environmental circumstances with mental and behavioral disorders (MBD) have been carried out, especially in developing countries such as Brazil.

**Methods:**

A time series study was carried out to explore the associations between daily air pollutants (SO_2_, NO_2_, O_3_, and PM_10_) concentrations and meteorological variables (temperature and relative humidity) on hospital admissions for mental and behavioral disorders for Curitiba, Brazil. Daily hospital admissions from 2010 to 2016 were analyzed by a semi-parametric generalized additive model (GAM) combined with a distributed lag non-linear model (DLNM).

**Results:**

Significant associations between environmental conditions (10 μg/m^3^ increase in air pollutants and temperature °C) and hospitalizations by MBD were found. Air temperature was the environmental variable with the highest relative risk (RR) at 0-day lag for all ages and sexes analyzed, with RR values of 1.0182 (95% CI: 1.0009–1.0357) for men, and 1.0407 (95% CI: 1.0230–1.0587) for women. Ozone exposure was a risk for all women groups, being higher for the young group, with a RR of 1.0319 (95% CI: 1.0165–1.0483). Elderly from both sexes were more susceptible to temperature variability, with a RR of 1.0651 (95% CI: 1.0213–1.1117) for women, and 1.0215 (95% CI: 1.0195–1.0716) for men.

**Conclusions:**

This study suggests that temperatures above and below the thermal comfort threshold, in addition to high concentrations of air pollutants, present significant risks on hospitalizations by MBD; besides, there are physiological and age differences resulting from the effect of this exposure.

**Graphical abstract:**

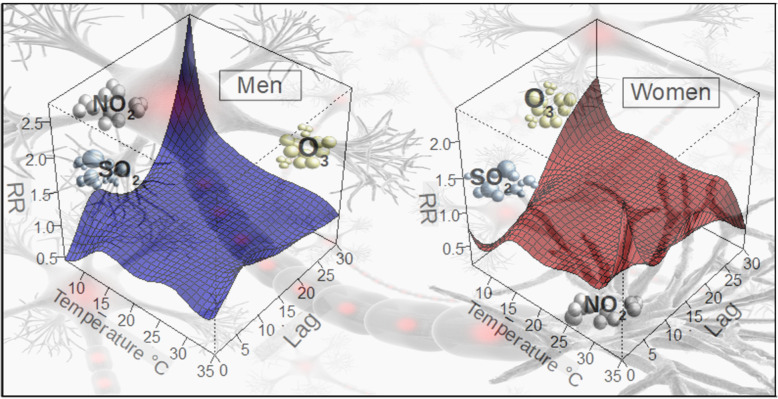

## Highlights

The highest relative risks occurred in the same day of the exposure (lag = 0).

Women are more affected by air pollutants and temperature variability than men.

Elderly from both sexes are more vulnerable to temperature variability.

Ozone exposure is a risk for women and higher for young group with MBD.

## Background

There are several strong evidences concerning the negative impacts of air pollution and extreme temperature on human health [1–3]. The complex mixture of chemical substances in polluted air associated with meteorological variables imposes various damages on circulatory and respiratory human system, as well as causes aggravation of diseases [4]. In addition, more recently, negative effects of these variables on mental disorders have been reported [5–8], including both Alzheimer’s and Parkinson’s diseases, which are progressive neurodegenerative diseases [9–14].

The mechanism of action is not completely understood, as well as the damages caused to immune system [15, 16]. However, there are evidences that men and women present brain differences, which are related to biological factors (genetic and hormonal signals), physical environment, and sociocultural experiences [17, 18].

Epidemiological studies concerning the mental diseases associations with air pollution and meteorological variables have been conducted in the last years for short-term [6, 19–22] and long-term exposure [23, 24]. Almendra [20] found that the hospital admissions by mental disorders increase with high temperatures, and they pointed out that low temperatures can be a protective factor for Lisbon population [25]. observed associations between traffic-related air pollution and dementia incidence in Northern Sweden.

For developing countries located in the South Hemisphere the relationship between mental disorders, air pollution and temperature has been scarcely explored. In Brazil, epidemiological studies investigating the potential associations and their behavior for sex and age groups are non-existent [26]. conducted a study to examine the association between meteorological factors and suicide in São Paulo, Brazil, and they suggested that a temperature increase has a short-term effect on suicide. According to [27], the prevalence of dementia among Brazilian population ranged from 5.1 to 17.5%, and the costs of dementia were estimated at US$16,548.24 per patient/year [28]. In addition, in Brazil life expectancy is increasing, in 2018 it was around 75 years and for 2050 is estimated to be 80 years [29]. Therefore, with increasing life expectancy and the inversion of the country’s age pyramid (around 9% in 2018 to 22% in 2050 of elderly, > 65 years), the fraction of population with dementia could be also increase, making it important to know the risks of environmental variables on development of these diseases, in order to provide subsidies for public health policies.

Thus, the aim of this study is to assess the short-term effects of daily environmental conditions (SO_2_, NO_2_, O_3_ and PM_10_, temperature and relative humidity) on hospitalizations for mental and behavioral disorders in Curitiba city, Paraná state, Brazil. The data was analyzed by a semi-parametric generalized additive model (GAM) combined with a distributed lag non-linear model (DLNM) for age groups, both sexes, and lag time (0–7 days).

## Methods

### Location

**Fig. 1:**
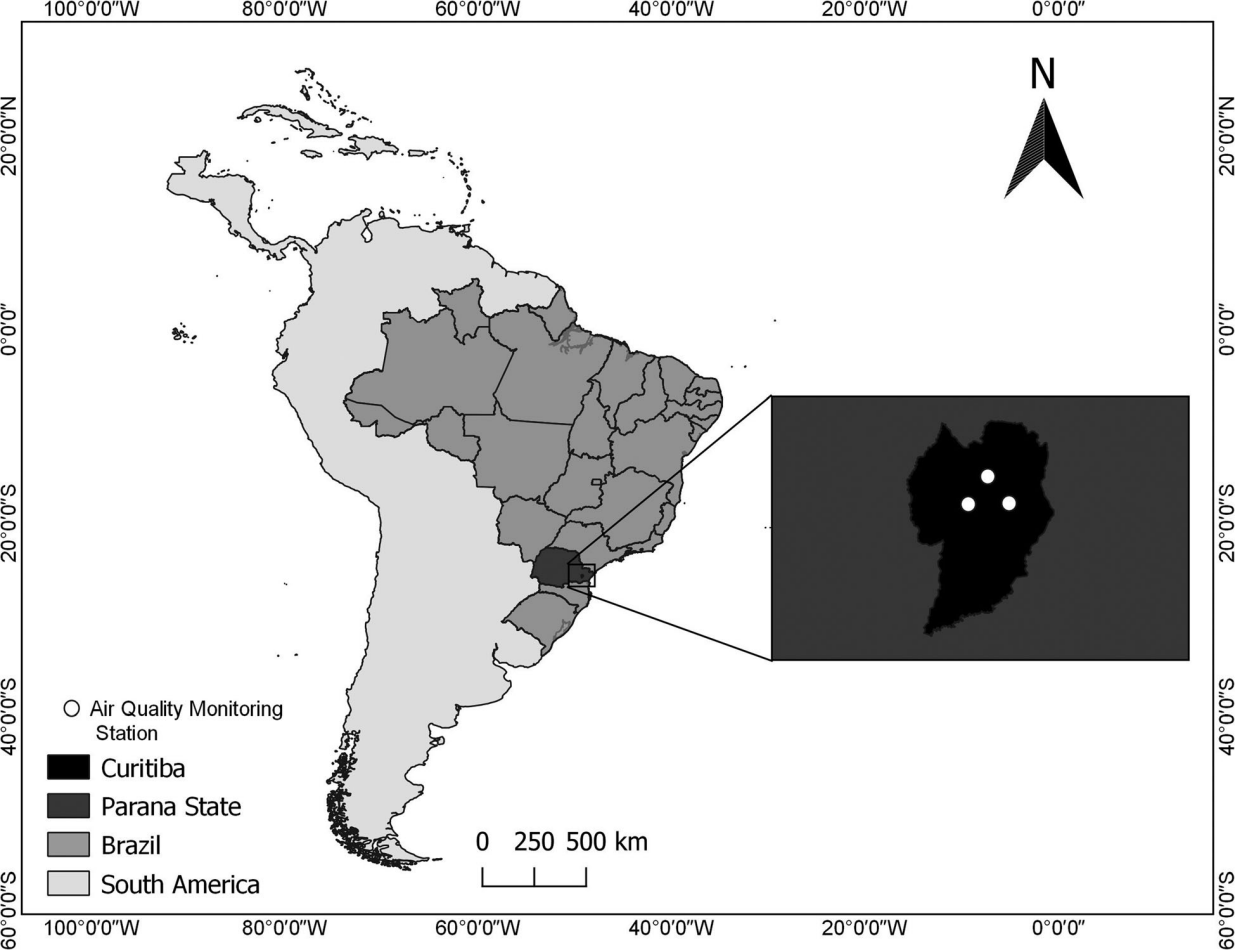
Area of study

The city of Curitiba belongs to a regional political and socioeconomic division of the Parana state, in Brazil’s southern region (Fig. 1). It is the main and most developed economic center of the state, and one of the most important in the country [29, 30]. The city is highly urbanized and has around 1,917,185 inhabitants, of whom approximately 97% reside in urban areas. Curitiba has its main economic activity in commerce, therefore the vehicular emission is the predominant source of pollution [30]. Particular attention is paid to Curitiba because it is known for its urban planning, which has attracted worldwide attention as an example of sustainable urban development [31].

The altitude of the region is near 900 m, with the climate classified according to W. Koeppen as tropical altitude of Cfa and Cfb [32]. The warmest months range from December to February with average daily temperatures between 17 °C to 23 °C, when the solar incidence in the region is more pronounced, and there is a predominance of intertropical atmospheric systems. The coldest season is concentrated between the months of June and August, when polar atmospheric systems are more present and the solar incidence is lower, with temperatures average around 13 °C. Rain occurs throughout the year, with less intensity in winter, which makes this season unfavorable for the dispersion of atmospheric pollutants [33–35].

### Data collection

The daily average time series from 2010 to 2016 of SO_2_, NO_2_, O_3_ and PM_10_ concentrations, as well as temperature and relative humidity were analyzed in all hospital admissions for mental and behavioral disorders (MBD). The daily number of hospitalizations for primary diagnosis of MBD (Chapter V, codes F00-F99) classified according to the International Classification of Diseases (ICD 10) were compiled from the website of public Single System of Health (SUS), Department of Informatics (DATASUS) webpage (www.datasus.saude.gov.br), as shown in Table 1.

**Table 1:** International Classification of Disease (ICD 10) codes for mental disorders used in the present study

ICD 10	Description
F00 - F09	Organic Mental Disorders, including Symptomatic Mental Disorders
F10 - F19	Mental and behavioral disorders due to the use of psychoactive substance
F20 - F29	Schizophrenia, schizotypal disorders and delusional disorders
F30 - F39	Mood Disorders [affective]
F40 - F48	Neurotic disorders, stress-related disorders and somatoform disorders
F50 - F59	Behavioral syndromes associated with physiological dysfunctions and physical factors
F60 - F69	Adult personality and behavior disorder
F70 - F79	Mental retardation
F80 - F89	Psychological developmental disorders
F90 - F98	Psychological developmental disorders
F99	Mental disorder not specified

The meteorological variables and air pollutant concentrations were obtained from the public Environmental Institute of Paraná (IAP – in Portuguese *Instituto Ambiental do Paraná*, www.iap.pr.gov.br). Three urban air quality automatic stations, located in Curitiba city (Fig. 1), were considered to calculate the daily means from hourly concentrations of pollutants and meteorological variables. The stations used had at least 80% of valid data.

### Statistical method

Firstly, a descriptive analysis was performed for the data. Then, the covariates were selected to compose the final analysis for each group by *Stepwise* method, which assists to identify the predictors to be inserted in the final model. The process verifies the significance of the explanatory variables by systematically adding the most significant variable or by removing the least significant one during each step, with inference based on the significance level of 5% [36, 37]. All variables were tested and only those with statistical significance were included in the final model (see the supplementary material). The analysis was stratified by sex and age group as: young from 0 to 24 years old, adults from 25 to 59 years old, and elderly with 60 years or more. These groups were defined according to changes of physiological characteristics due to the aging of the organism [38–40].

It was used a generalized additive model (GAM) combined with a distributed lag non-linear model (DLNM), a modeling framework that can simultaneously represent non-linear exposure-response dependencies and delayed effects [41, 42]. The combined use of the DLNM model provides values of the effect of the event with *N*-day lag, also emphasizing the cumulative effect measurement during the period [41, 42]. For the assessment of short-term exposure to air pollutants and meteorological variables, the established delay was from 0 to 7 days [19, 43, 44].

Weekdays and holidays were added to the model as a factor variable with 7 levels (Monday to Sunday) in the case of days of the week, and a binary indicator (0 or 1) for the holidays, to soften the characteristics of hospitalizations concentrated on certain days of the week [33]. The meteorological and air quality variables, due to their seasonality, were modeled by natural cubic splines with 3 degrees of freedom (*df*) for pollutants and relative humidity, 5 *df* for temperature and 7 *df* per year for time space [19, 21, 43–48]. In this study, the number of the MBD is the random variable denoted by *Y*_*i*_*(i = 1, …, n),* and the distribution of *Y*_*i*_ in a GAM may be any distribution belonging to the exponential family [49]. Among the GAM, two distributions were tested: Poisson and negative binomial, which are widely used distributions in epidemiological studies involving counting data. The latter one distribution have a parameter that corrects the overdispersion of the data, which is expected in this type of study [19, 33, 50]. It was also considered an extension of the Poisson distribution, called the quasi-Poisson model, including of a dispersion parameter [51]. For the systematic part [42, 52], the semi-parametric model is given by:

$$ g\left({\mu}_i\right)=\alpha +\sum \limits_{j=1}^6{s}_j\ \left({x}_{ji}\right)+{\beta}_1{day}_i+{\beta}_2{holy}_i+{\beta}_3\  time,i=1,\dots, n, $$

where *E*(*Y*_*i*_) = *μ*_*i*_, *g(.)* is a logarithmic link function, α is the intercept, *s(.)* represents the natural cubic spline for non-linear predictor variables, *x*_*ji*_ denotes the explanatory variables (daily mean temperature, relative humidity and pollutant concentrations), *time* refers to the effect of the temporal trend, *day* is the day of the week parameter and finally *holy*, which refers to holidays.

The relative risk (RR) with 95% confidence interval (CI) of hospital admissions for MBD were calculated for significant variables (*p*-value < 0.05) for each age group to the cumulative effect of 0 to 7 days exposure. Statistical analyzes were performed using the statistical software R 3.5.3 with the “DLNM” package.

## Results

### Exploratory analysis

Table 2 presents descriptive statistics for all daily variables analyzed. Of the 5397 hospitalizations recorded in the study period (2010–2016), approximately 60% were of men. On average, there were three daily hospital admissions by MBD. Regarding environmental conditions, the mean daily temperature was 22.4 °C, ranging from 5.9 to 35.0 °C, the mean daily relative humidity was 81.8%, and the mean daily concentrations for SO_2_, NO_2_, O_3_ and PM_10_ were of 0.53 μg/m^3^, 32.1 μg/m^3^, 22.7 μg/m^3^ and 10.9 μg/m^3^, respectively.

**Table 2:** Summary of daily hospital admissions for mental and behavioral disorders, meteorological variables and air pollutants in Curitiba, 2010–2016

Variable	Unit	Minimum	25th	50th	75th	Maximum
SO_2_	*μ*g/m^3^	0	0.05	2.7	0.5	188.2
NO_2_	*μ*g/m^3^	5.0	23.5	39.2	32.1	114.2
O_3_	*μ*g/m^3^	0.3	14.4	28.3	22.7	81.4
PM_10_	*μ*g/m^3^	1.3	6.9	18.7	10.9	116.0
T	^*°*^C	5.9	18.4	26.2	22.4	35.0
RH	%	39.0	75.5	87.8	81.8	99.9
Men	hosp.	0	1	2	2	29
Women	hosp.	0	0	1	1	20
Young men	hosp.	0	0	1	1	12
Adult men	hosp.	0	2	3	2	27
Elderly men	hosp.	0	0	1	1	6
Young women	hosp.	0	1	1	2	5
Adult women	hosp.	0	1	2	2	19
Elderly women	hosp.	0	0	1	1	6

The final fit of the model was assessed using the Akaike information criterion (AIC) [53], and the residual deviance [54, 55]. For Poisson distribution the AIC was of 6717.3 and residual deviance of 4436.7. The quasi-Poisson did not estimate AIC values, but the residual deviance was 4436.7. While the negative binomial presented AIC of 5291.9 and residual deviance of 1546.3. Therefore, the distribution that fitted the dataset was the negative binomial, which was used for the analyses.

Table 3 presents the results of the *Stepwise* method for each group, and their respective variables included in the final analysis. Additional results from the application of *Stepwise* method are presented as supplementary material. Considering the *p*-value < 0.05 criterion, the PM_10_ concentration was significant only for groups of men, except for the elderly group. Fine particle concentrations are not measured regularly in the city, therefore not analyzed. In Brazil, only in the year 2018 the PM_2.5_ was included in the national air quality standard (60 μg/m^3^, 24 h).

**Table 3:** Variables selected by *Stepwise* method (significance level *α* = 0.05) to compose the final model of each group

Groups	Variables	***p***-value
Men	T, SO_2_, NO_2_, O_3_, PM_10_	*<* 0.0001
Women	T, SO_2_, O_3_	*<* 0.0001
Young men (0–24 years old)	T, SO_2_, O_3_, PM_10_	*<* 0.0001
Adult men (25–59 years old)	T, SO_2_, NO_2_, O_3_, PM_10_	*<* 0.0001
Elderly men (≤ 60 years old)	T, SO_2_, O_3_	*<* 0.0001
Young women (0–24 years old)	T, SO_2_, O_3_	*<* 0.0001
Adult women (25–59 years old)	T, SO_2_, O_3_	*<* 0.0001
Elderly women (≤ 60 years old)	T, SO_2_, O_3_	*<* 0.0001

### Distributed lagged effects

The GAM with random component given by negative binomial distribution was the one which best fitted the hospital admissions by MBD. The most significant RR was found in the lag 0 day for all groups analyzed, which means that environmental conditions rapidly affect individuals with MBD.

Table 4 shows the significant cumulative effects (0–7 days) of environmental variables on hospital admissions by MBD for men and women, which were adjusted according to the temporal trend, day of the week, relative humidity and air pollutant concentrations for each selected group. For men, lag 0 and lag 0–7 presented risk of exposure to environmental conditions, corresponding to 1.1911 (95% CI: 1.1459–1.2399), and 1.0841 (95% CI: 1.0816–1.1266), respectively. For women, the lag 0, lags 0–6 and 0–7 also presented risks, although their values were lower. At lag 0 the RR value was 1.1891 (95% CI: 1.1517–1.2290), and for the lag 0–6 and lag 0–7 were of 1.0647 (95% CI: 1.0321–1.0992) and 1.0816 (95% CI: 1.0487–1.1167), respectively.

**Table 4:** Relative risks with 95% confidence interval (in parentheses) of hospital admissions due to mental and behavioral disorders associated with daily mean temperature vs reference temperature (22.4 °C) and air pollutants concentrations by sex

–	Men	Women
Lag 0	1.1911 (1.1459–1.2399)	1.1891 (1.1517–1.2290)
Lag 0–6	–	1.0647 (1.0321–1.0992)
Lag 0–7	1.0841 (1.0446–1.1266)	1.0816 (1.0487–1.1167)
NO_2_^a^	1.0175 (1.0105–1.0246)	–
SO_2_^a^	–	1.0121 (1.0029–1.0123)
O_3_^a^	–	1.0161 (1.0087–1.0236)
T^a^	1.0182 (1.0009–1.0357)	1.0407 (1.0230–1.0587)

^a^Relative risk at lag 0

In the final multivariable model of women’s group, only the temperature, SO_2_ and O_3_ variables were selected by applying the *Stepwise* method. However, the pollutants were also a risk for MBD at lag 0, showing an increase of 1.2 and 1.6%, respectively for SO_2_ and O_3_ in hospitalizations by MBD. In case of temperature, the RR value was 1.0407 (95% CI: 1.0230–1.0587) for women.

For men, despite the range of pollutants selected by the model, only NO_2_ present a RR of 1.0175 (95% CI: 1.0105–1.0246) in lag 0, besides air temperature (RR 1.0182, 95% CI: 1.0009–1.0357). In contrast, the women’s group presented a risk associated with all pollutants selected for the multivariable model, as presented in Table 4.

The values of RR for groups stratified by sex and age are show in Table 5. The environmental conditions presented pronounced RR at lag 0 day for all age groups (young, adult and elderly), and sexes. The RR values for men’s groups were higher than women, except for the young group. For young women group the RR was of 1.1987 (95% CI: 1.1191–12,892) at lag 0 day, while for young men group was of 1.1400 (95% CI: 1.0551–1.2366). Among age groups, the adult group was susceptible to air pollutants and temperature variation, with women groups being more affected by air pollutants than men. Ozone was a risk for all women groups, and even higher for the young group, with RR of 1.0319 (95% CI: 1.0165–1.0483).

**Table 5:** Relative risks with 95% confidence interval (in parentheses) of hospital admissions due to mental and behavioral disorders associated with daily mean temperature vs reference temperature (22.4 °C) and air pollutants concentrations stratified by sex and age

	**Young men**	**Adult men**	**Elderly men**
Lag 0	1.1400 (1.0551–1.2366)	1.2102 (1.1573–1.2679)	1.1877 (1.0548–1.3559)
Lag 0–1	–	1.0496 (1.0056–1.0969)	–
Lag 0–7	–	1.0864 (1.0397–1.1371)	1.1306 (1.0032–1.2868)
NO_2_^a^	–	1.0181 (1.0101–1.00263)	–
T	1.0133 (1.0105–1.0479)	1.0225 (1.0025–1.0428)	1.0215 (1.0195–1.07161)
–	**Young women**	**Adult women**	**Elderly women**
Lag 0	1.1987 (1.1191–1.2892)	1.1904 (1.1499–1.2337)	1.1209 (1.0348–1.2184)
Lag 0–1	–	1.0419 (1.0047–1.0818)	–
Lag 0–6	–	1.0683 (1.0325–1.1063)	–
Lag 0–7	–	1.0829 (1.0472–1.1212)	1.0993 (1.0207–1.1867)
SO_2_^a^	–	1.0126 (1.0024–1.0249)	–
O_3_^a^	1.0319 (1.0165–1.0483)	1.0241 (1.0169–1.0315)	1.0198 (1.0034–1.0367)
T^a^	1.0598 (1.0195–1.1027)	1.0543 (1.0374–1.0717)	1.0651 (1.0213–1.1117)

^a^Relative risk at lag 0

**Fig. 2:**
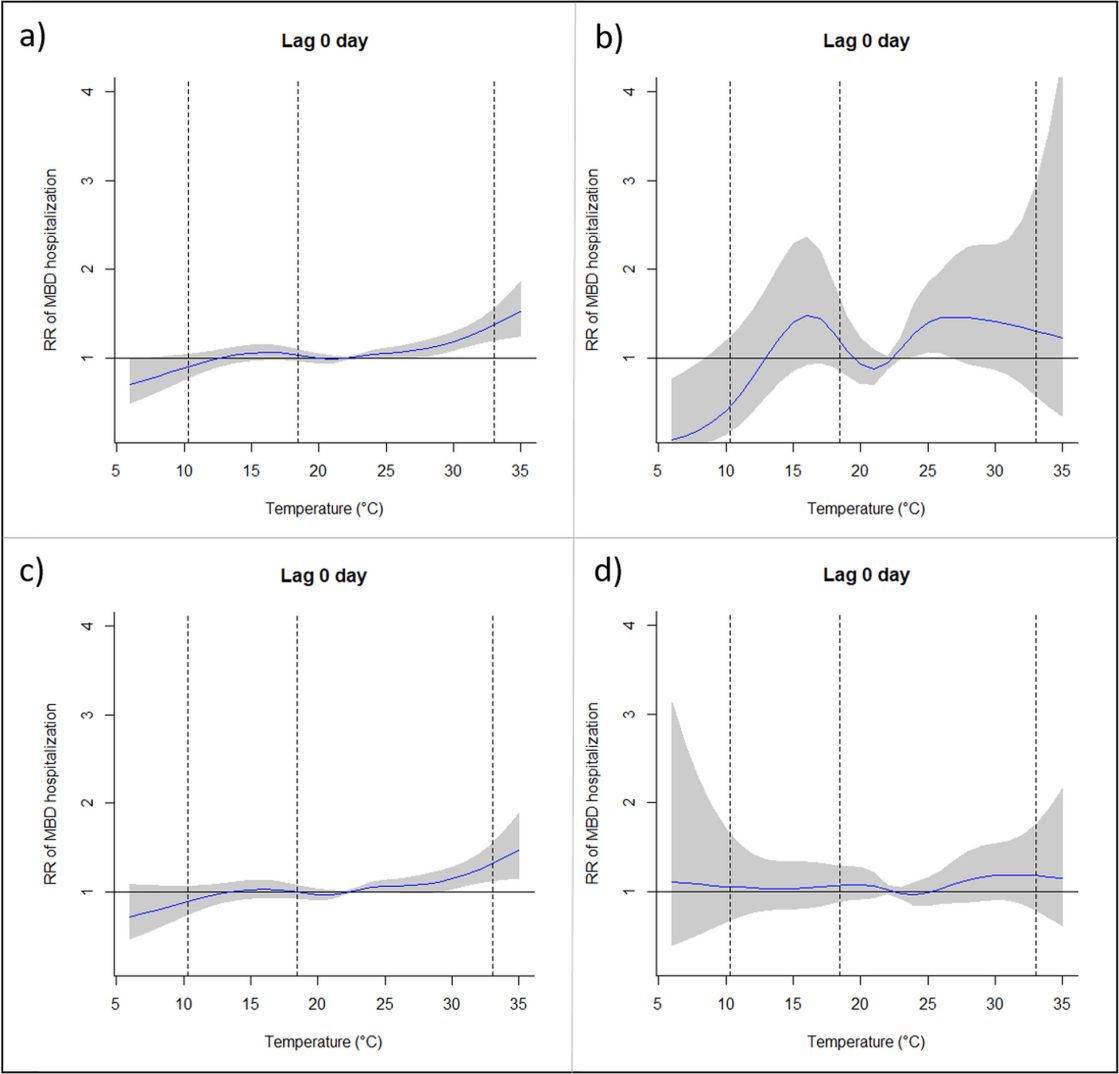
Exposure-response curve of mean temperature (°C) and relative risk for mental and behavioral disorder (reference temperature at 22.4 °C) for men. a. Group of all men. b. Group of young men. c. Group of adult men. d. Group of elderly men. Vertical lines mean the 1st, 25th and 99th percentiles

**Fig. 3:**
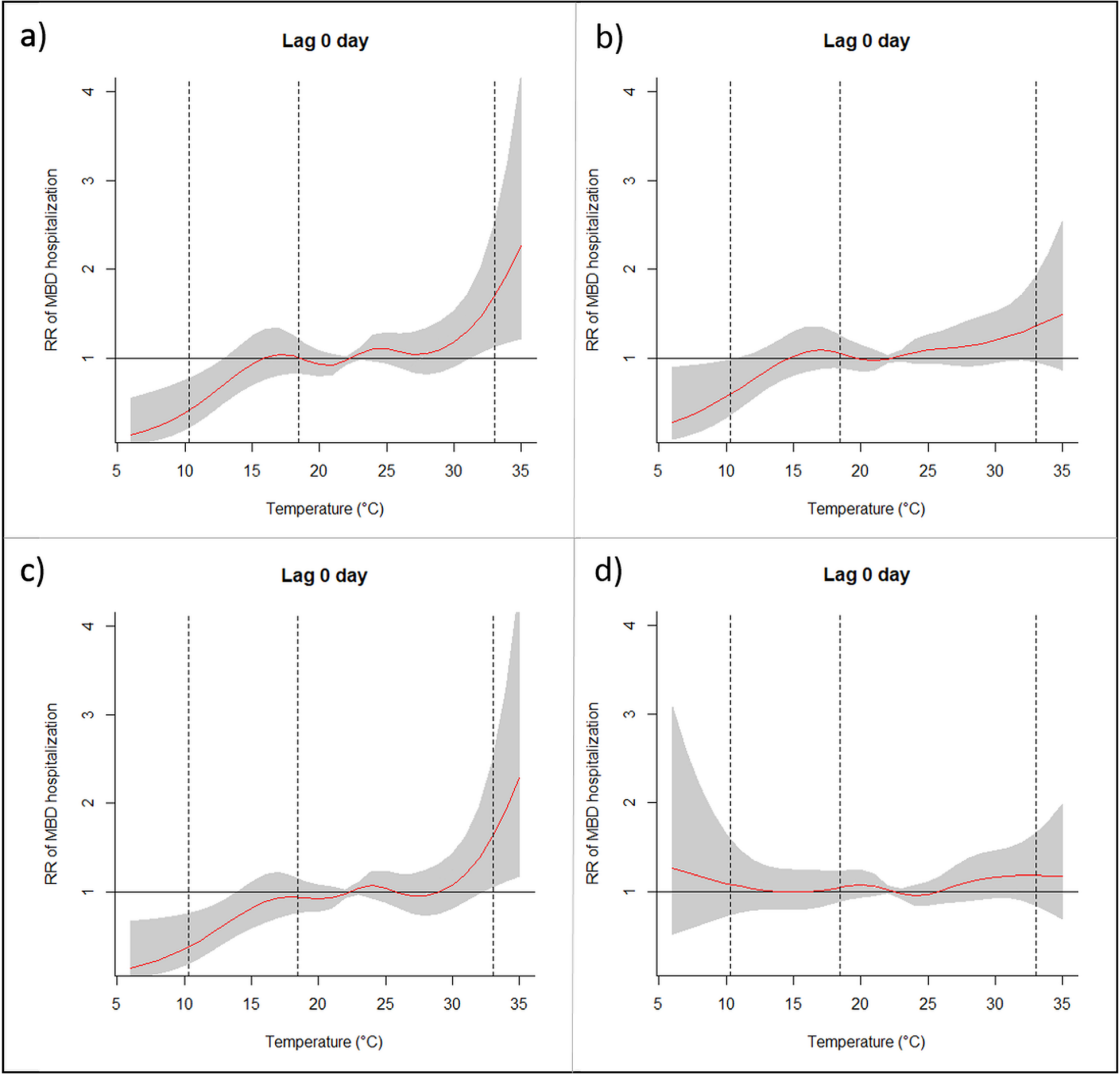
Exposure-response curve of mean temperature (°C) and relative risk for mental and behavioral disorder (reference temperature at 22.4 °C) for women. a. Group of all women. b. Group of young women. c. Group of adult women. d. Group of elderly women. Vertical lines mean the 1st, 25th and 99th percentiles

Air temperature was the environmental variable with highest RR at lag 0 day for all ages and sexes analyzed. Therefore, the behavior of RR as a function of temperature was deeply analyzed. Figures 2 and 3, for men and women, respectively, show the exposure-response curves of mean temperature and the significant risks on hospital admissions by MBD for all groups analyzed at lag 0 day. The exposure-response curves were different among groups of men analyzed. In the young group, significant RR were found for temperatures above 22.4 °C, and also for lower temperatures, with a peak risk at 15.7 °C. On the other hand, for adult group significant RR were found only for elevated temperatures above 22.4 °C (percentile 50th), while for elderly group air temperature was a risk for almost the entire range, except around mean daily temperature. The groups of young and elderly men presented significant differences in the behavior and values, when compared with the curve for all men (Fig. 2a), indicating the relevance of age on hospital admissions by MBD, with young and elderly groups being the most affected.

For the women groups the exposure-response curves also presented differences, although they were less pronounced when compared with men. For elderly group air temperature represented a risk for almost the whole range, except around mean daily temperature, while for all women and adult group the risk increases significantly in case of extremely high temperatures, above 30 °C (Fig. 3a and c).

## Discussion

In Brazil, there are several studies addressing the association between hospitalization and deaths by cardiorespiratory diseases with air quality and meteorological variables [56–58]. However, concerning the hospital admissions by mental and behavioral disorders, and their association with environmental conditions (air pollution and temperature) the studies are very limited. To our knowledge, this is the first epidemiological study addressing the association between temperature and air pollutants concentrations with hospital admissions for MBD in Brazil.

The cumulative short-term effects of air pollutants, temperature and relative humidity on hospitalization by MBD for men and women groups were investigated in this work. The quasi-Poisson model is widely used [19, 21, 22], however, in our analysis and based on AIC results, a best fit to dataset was obtained by the Negative Binomial model, as found by [33]. The different models used in the studies available in the literature make it difficult to compare the results, although it is important to evaluate the adequacy of the model to the dataset, which could lead to misinterpretation of results, such as overestimation of RR.

Montero [59] emphasize the importance to analyze separately temperature and relative humidity, since these variables are correlated. Relative humidity did not present any risk in this study, probably because there was a slight variation of this variable over the period. Therefore, due to its low variability, humidity is rarely the explicit focus in health impact studies, despite its physiological importance [60, 61]. In the same way, other environmental variables could be correlated, and should be corrected, this potential multicollinearity, as performed in this and previous works [21, 62].

We found significant impacts of environmental variables on hospitalization by MBD, with differences between men and women, as well as among different age groups. At lag 0 the RR for all environmental variables were higher for men than women, except for young group [63]. observed a greater number of significant associations for women than men exposed to gaseous pollutants, as obtained in the present study. PM_10_ concentrations were not statistically significant and therefore were not included in the final model for women. It is important to note that fine particles concentrations could not be analyzed, which are those with stronger evidences of causality [22, 64, 65].

Air pollution may exert adverse effects on central nervous system function [12], with men and women reacting differently to this exposure [66]. For example [67], ,have shown that exposure to O_3_ is associated with the reduced performance of cognitive activities, such as short-term memory and coding skills. This is consistent with our observations that O_3_ was found as a significant variable, and also a risk for women. Elevation of the level of free radicals related to O_3_ exposure to affects neurodegenerative processes causing oxidative stress, which alters the brain structures [68, 69].

Lin [70] also observed the influence of sex on sensitivity to types of pollutants, i.e., the men exhibited sensitivity to particulate matter, while women to NO_2_; and proposed, as an explanation, the difference on physiological characteristics. The behavior of each gender is also an important factor, since the activities of each individual (for example, cooking, cleaning, lawn care) produce different exposure patterns [71]. In contrast [6], did not found significant differences in the association between sex and age groups for Shanghai, China.

Studies concerning neurodegenerative disorders indicated difference on prevalence and severity of these disorders between the two sexes [17, 18, 72, 73]. Ullah [18] pointed out that these disorders are influenced by genetic and lifestyle factors, concurrent health conditions as well as un-modifiable predisposing risk factors, including gender and age.

Among environmental variables analyzed (O_3_, NO_2_, SO_2_, PM_10_, temperature and relative humidity), air temperature presented the highest RR for hospitalization by MBD at lag 0. The RR for temperature at lag 0 day was higher for women than men (RR at Table 5), as also found by [20], and suggested by [19]. We found differences among age groups analyzed, with elderly being more affected and higher RR to temperature exposure at lag 0.

The exposure-response curves of temperature in this work differ from those obtained for Lisbon [20]. Our results, analyzing also the cumulative effects, indicated that hospital admissions increase significantly with high and also low temperatures, especially for young men group (the curves for all significant lags and considering all groups are provided as supplementary material). In addition, for elderly groups temperature is a risk for almost all range, except around comfortable temperature, i.e., the mean daily temperature (22.4 °C). Biological factors can explain this behavior, since the thermoregulatory system with ageing decreases the ability to regulate the core body temperature [74, 75].

## Conclusions

The present study showed significant associations between the environmental conditions (SO_2_, NO_2_, O_3_ and PM_10_, temperature and relative humidity) and hospitalizations by mental and behavioral disorders. The effects were immediate and non-linear. Temperature was the variable responsible for the most pronounced increase in the risk of hospital admissions by MBD, affecting men and women differently, as well as the age groups.

The synergistic association of air pollutants and temperature affected more men than women, except for the young group of men. Among age groups, the adult group was more susceptive to air pollutants and temperature, with women being more affected by air pollutants than men. In addition, the individual effect of temperature was more pronounced among women of all age groups. Finally, the elderly of both sexes were the most susceptible to temperature variability. From results obtained in this study, it can be pointed out the necessity of public policies to control the atmospheric emissions, as well as for adaptation to global climate change.

## Supplementary information

**Additional file 1 **: **Figure S1:** Exposure-response curve of ean temperature (°C) ad cumulative relative risk for mental and behavioral disorder (reference temperature at 22.4 °C) for men. a. Group of all men. b. Group of young men. c. Group of adult men. d. Group of elderly men. Vertical lines correspond to the 1st, 25th and 99th temperature percentiles. **Figure S2** Exposure-response curve of mean temperature (°C) and cumulative relative risk for mental and behavioral disorder (reference temperature at 22.4 °C) for women. a. b. Group of all women. c. d. e. Group of adult women. f. Group of elderly women. Vertical lines correspond to the 1st, 25th and 99th temperature percentiles. The Stepwise method is a procedure for selecting or deleting variables from a model. It is based on an algorithm that checks the importance of variables, including or excluding them from the model based on a decision rule. The importance of the variable is defined in terms of a measure of statistical significance of the coefficient associated with the variable for the model. **Table S1***P*-value used by the method (significance level α = 0.05) for selection of variables in the final model of each group.

## Data Availability

The dataset used and/or analyzed in this study are available online or under requested at public institutions: Datasus (www.datasus.saude.gov.br) and IAP (www.iap.pr.gov.br).

## Abbreviations

AIC: Akaike information criterion
CAPES: Coordenação de Aperfeiçoamento de Pessoal de Nível Superior
CI: Confidence interval
CNPq: Conselho Nacional de Desenvolvimento Científico e Tecnológico
DATASUS: Computer Science Department of the Unified Health System
df: degrees of freedom
DLNM: Distributed lag non-linear model
GAM: Generalized additive model
IAP: Environmental Institute of Parana
ICD: Internal Classification of Diseases
MBD: Mental and behavioral disorders
NO_2_: Nitrogen dioxide
O_3_: Ozone
PM_10_: Particulate material 10
PM_2.5_: Particulate material 2.5
RR: Relative risk
SO_2_: Sulfur dioxide
SUS: Single System of Health
