# Supplementary material for: Risk assessment of temperature and air pollutants on hospitalizations for mental and behavioral disorders in Curitiba, Brazil
Source: Environ Health. 2020 Jul 6;19:79. doi: 10.1186/s12940-020-00606-w (PMC7336420; doi:10.1186/s12940-020-00606-w)
Supplement: Supplementary file 1 — Additional file 1 : Figure S1: Exposure-response curve of ean temperature (°C) ad cumulative relative risk for mental and behavioral disorder (reference temperature at 22.4 °C) for men. a. Group of all men. b. Group of young men. c. Group of adult men. d. Group of elderly men. Vertical lines correspond to the 1st, 25th and 99th temperature percentiles. Figure S2 Exposure-response curve of mean temperature (°C) and cumulative relative risk for mental and behavioral disorder (reference temperature at 22.4 °C) for women. a. b. Group of all women. c. d. e. Group of adult women. f. Group of elderly women. Vertical lines correspond to the 1st, 25th and 99th temperature percentiles. The Stepwise method is a procedure for selecting or deleting variables from a model. It is based on an algorithm that checks the importance of variables, including or excluding them from the model based on a decision rule. The importance of the variable is defined in terms of a measure of statistical significance of the coefficient associated with the variable for the model. Table S1P-value used by the method (significance level α = 0.05) for selection of variables in the final model of each group. [file 12940_2020_606_MOESM1_ESM.docx]

Supplementary material


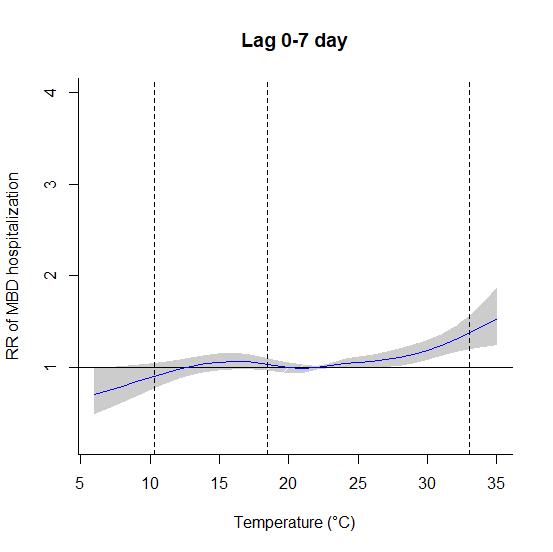

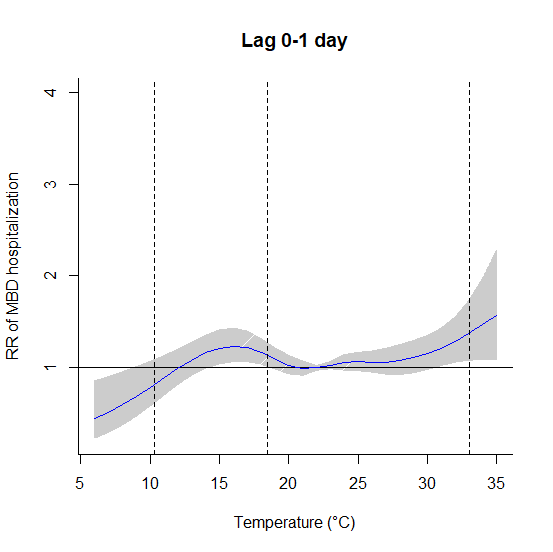

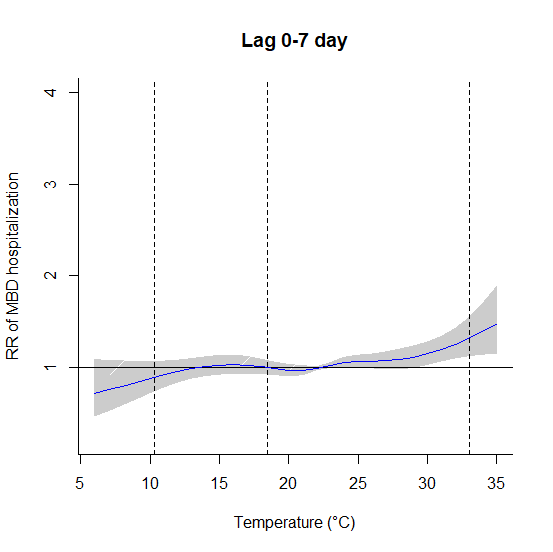

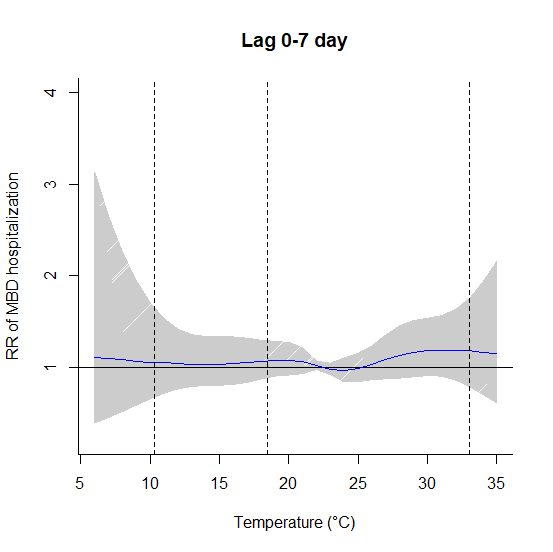


c)

d)

b)

a)

**Figure S1:** Exposure-response curve of mean temperature (°C) and cumulative relative risk for mental and behavioral disorder (reference temperature at 22.4 °C) for men. a. Group of all men. b. Group of young men. c. Group of adult men. d. Group of elderly men. Vertical lines correspond to the 1st, 25th and 99th temperature percentiles.


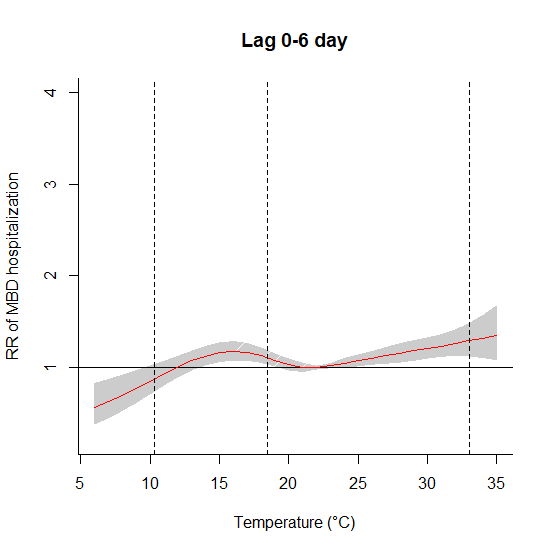

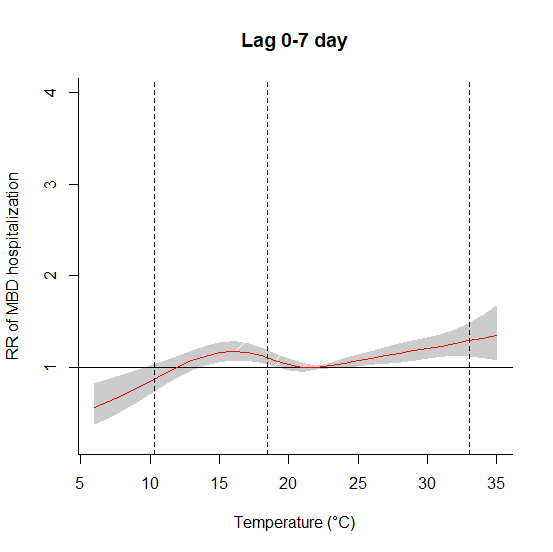

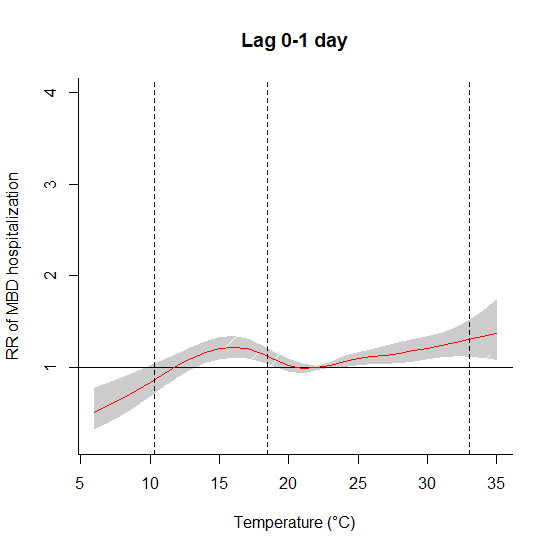

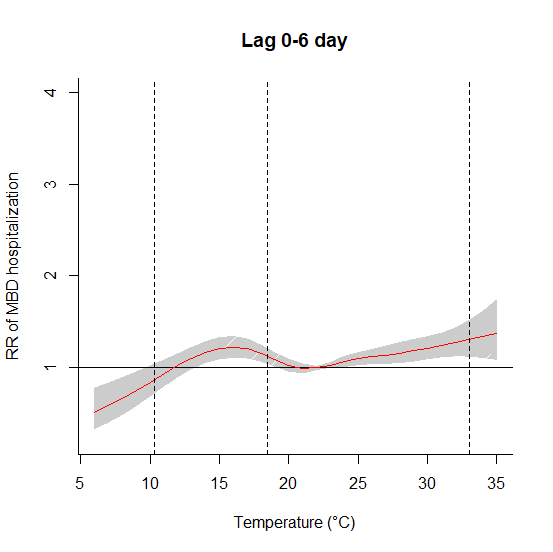

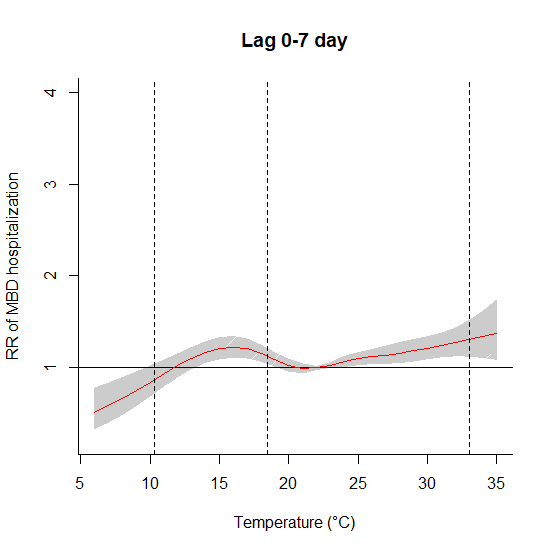

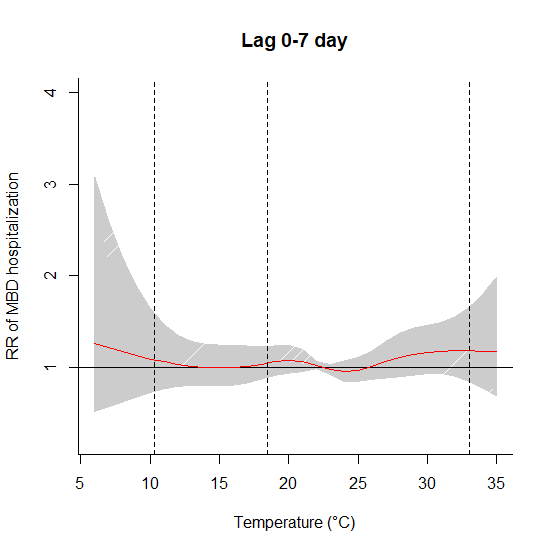


a)

c)

d)

e)

f)

b)

**Figure S2:** Exposure-response curve of mean temperature (°C) and cumulative relative risk for mental and behavioral disorder (reference temperature at 22.4 °C) for women. a. b. Group of all women. c. d. e. Group of adult women. f. Group of elderly women. Vertical lines correspond to the 1st, 25th and 99th temperature percentiles.

The *Stepwise* method is a procedure for selecting or deleting variables from a model. It is based on an algorithm that checks the importance of variables, including or excluding them from the model based on a decision rule. The importance of the variable is defined in terms of a measure of statistical significance of the coefficient associated with the variable for the model.

Table **S1:** *P-value* used by the method (significance level α = 0.05) for selection of variables in the final model of each group.

| **Groups** | **T** | **RH** | **SO_2_** | **NO_2_** | **O_3_** | **PM_10_** |
| --- | --- | --- | --- | --- | --- | --- |
| Men | ***<*0.0001** | 0.518 | ***<*0.0001** | ***<*0.0001** | ***<*0.0001** | ***<*0.0001** |
| Women | ***<*0.0001** | 0.517 | ***<*0.0001** | 0.528 | ***<*0.0001** | 0.971 |
| Young men (0-24 years old) | ***<*0.0001** | 0.497 | ***<*0.0001** | 0.692 | ***<*0.0001** | ***<*0.0001** |
| Adult men (25-59 years old) | ***<*0.0001** | 0.316 | ***<*0.0001** | ***<*0.0001** | ***<*0.0001** | ***<*0.0001** |
| Elderly men (≤ 60 years old) | ***<*0.0001** | 0.864 | ***<*0.0001** | 0.364 | ***<*0.0001** | 0.058 |
| Young women (0-24 years old) | ***<*0.0001** | 0.644 | ***<*0.0001** | 0.524 | ***<*0.0001** | 0.936 |
| Adult women (25-59 years old) | ***<*0.0001** | 0.923 | ***<*0.0001** | 0.887 | ***<*0.0001** | 0.516 |
| Elderly women (≤ 60 years old) | ***<*0.0001** | 0.517 | ***<*0.0001** | 0.528 | ***<*0.0001** | 0.971 |
